# Complex patterns of concomitant medication use: A study among Norwegian women using paracetamol during pregnancy

**DOI:** 10.1371/journal.pone.0190101

**Published:** 2017-12-28

**Authors:** Stefania Salvatore, Diana Domanska, Mollie Wood, Hedvig Nordeng, Geir Kjetil Sandve

**Affiliations:** 1 Department of Informatics, University of Oslo, Oslo, Norway; 2 PharmaTox Strategic Research Initiative, Faculty of Mathematics and Natural Sciences, University of Oslo, Oslo, Norway; 3 PharmacoEpidemiology and Drug Safety Research Group, School of Pharmacy, University of Oslo, Oslo, Norway; 4 Department of Child Health, Norwegian Institute of Public Health, Oslo, Norway; Karolinska Institutet, SWEDEN

## Abstract

**Background:**

Studies on medication safety in pregnancy often rely on an oversimplification of medication use into exposed or non-exposed, without considering intensity and timing of use in pregnancy, or concomitant medication use. This study uses paracetamol in pregnancy as the motivating example to introduce a method of clustering medication exposures longitudinally throughout pregnancy. The aim of this study was to use hierarchical cluster analysis (HCA) to better identify clusters of medication exposure throughout pregnancy.

**Methods:**

Data from the Norwegian Mother and Child Cohort Study was used to identify subclasses of women using paracetamol during pregnancy. HCA with customized distance measure was used to identify clusters of medication exposures in pregnancy among children at 18 months.

**Results:**

The pregnancies in the study (N = 9 778) were grouped in 5 different clusters depending on their medication exposure profile throughout pregnancy.

**Conclusion:**

Using HCA, we identified and described profiles of women exposed to different medications in combination with paracetamol during pregnancy. Identifying these clusters allows researchers to define exposure in ways that better reflects real-world medication usage patterns. This method could be extended to other medications and used as pre-analysis for identifying risks associated with different profiles of exposure.

## 1. Introduction

Over the past decade, increasing prevalence of medication use during pregnancy has led to a greater demand for studies investigating the safety of these medicines for the pregnant women and the developing fetus. However, there is not yet a consensus on how medication exposure should be defined. Most studies of medication use during pregnancy use over-simplified definitions of exposure: most commonly, “ever exposed” versus “never exposed.” By equating exposure to a single dose of a medication to chronic use over many days, these exposure definitions ignore important aspects of exposure such as dosage of medication, treatment duration, and timing of exposure [1]. Some studies have attempted to address this problem by categorizing exposure in trimester categories [2], or defining exposure by examining the number of days of medication use or number of windows of exposure. Focusing on one aspect of exposure to the exclusion of others (e.g., focusing on timing and not intensity) is an improvement over simpler strategies, but may still elide important differences in exposure patterns. Moreover, finely dividing exposure groups into categories that fully describe both timing and intensity can result in sparse data, even in very large studies. One possibility for addressing this problem is using data reduction techniques to describe exposure patterns.

A recent study by Hurault-Delarue and co-authors [3] has proposed using unsupervised clustering via the k-means method to identify patterns of medication use in pregnant women to consider the individual medication exposure trajectory in pregnancy. The authors estimated Defined Daily Dose (DDD) [4] of each medication used by each mother during pregnancy from data retrieved from the French prescription database (EFEMERIS) and then clustered these trajectories by using an extension of the traditional k-means algorithm to longitudinal data. Using this method, in a second study, they successfully identified and compared different profiles of women exposed to medications during pregnancy [5]. However, the k-means approach is limited to interval scale quantitative variables, and cannot be applied for categorical variables [6], which limits its utility when data on dose intensity are not available. Another study has used latent class analysis (LCA) to include both medication use patterns and other clinical characteristics as predictors of negative outcomes in pharmacoepidemiological studies [7]. While LCA is a more flexible approach than k-means for the inclusion of variables of mixed scale types [6–8], it requires a data reduction technique prior to its application when the number of concomitant medications considered in the analysis is high. In this study, more than 360 distinct medications, corresponding to more than 1000 different concomitant medications, were detected, making the application of LCA less suitable.

We propose improving upon these approaches by using a different clustering method for better identifying high-risk exposure patterns. Hierarchical cluster analysis (HCA) is a flexible data reduction technique that allows for the use of categorical variables and longitudinal data. Indeed, by defining an appropriate distance measure, HCA can use any type of variable to create a hierarchy of clusters which can be visualized through a dendrogram. While the k-means procedure uses a simple distance vector towards centroids and LCA computes classification probabilities [7], HCA allows for the use of a custom distance metric, which can be used to define similarity between women according to the Anatomic Therapeutic Chemical (ATC) [9] classification code of the medications used in pregnancy.

Paracetamol exposure in pregnancy is a useful motivating example in this study, because it is a widely-used medication whose pattern of use differs greatly between women, and because any effects of exposure are likely highly dependent on dose and timing. Previous research into the safety of paracetamol in pregnancy suggests an increased risk of ADHD or other neurodevelopmental problems [10–15]. Our previous investigations into effects of prenatal paracetamol exposure have divided women into low-dose and high-dose users [14, 15] and found greater risks of neurodevelopmental problems in children with higher exposure, after adjusting for possible confounders; however, it is possible that exposure classification that better reflects realistic usage patterns can provide more relevant clinical information. This study used HCA to define longitudinal medication use patterns among pregnant women in a sub-population of the Norwegian Mother and Child Cohort Study (MoBa) of women using paracetamol.

## 2. Methods

### Data material

The Norwegian Mother and Child Cohort Study (MoBa) is a prospective population-based pregnancy cohort study conducted by the Norwegian Institute of Public Health. Participants were recruited from all over Norway from 1999–2008. The women consented to participation in 41% of the pregnancies. The cohort now includes 114 500 children, 95 200 mothers and 75 200 fathers [16]. MoBa have obtained a license from the Norwegian Data Inspectorate. The present study was approved by the Regional Committee for Medical Research Ethics. The current study is based on version 9 of the quality-assured data files released for research in 2016; these data were linked to the Medical Birth Registry of Norway (version 4.6.0 released in 2015).

**Fig 1:**
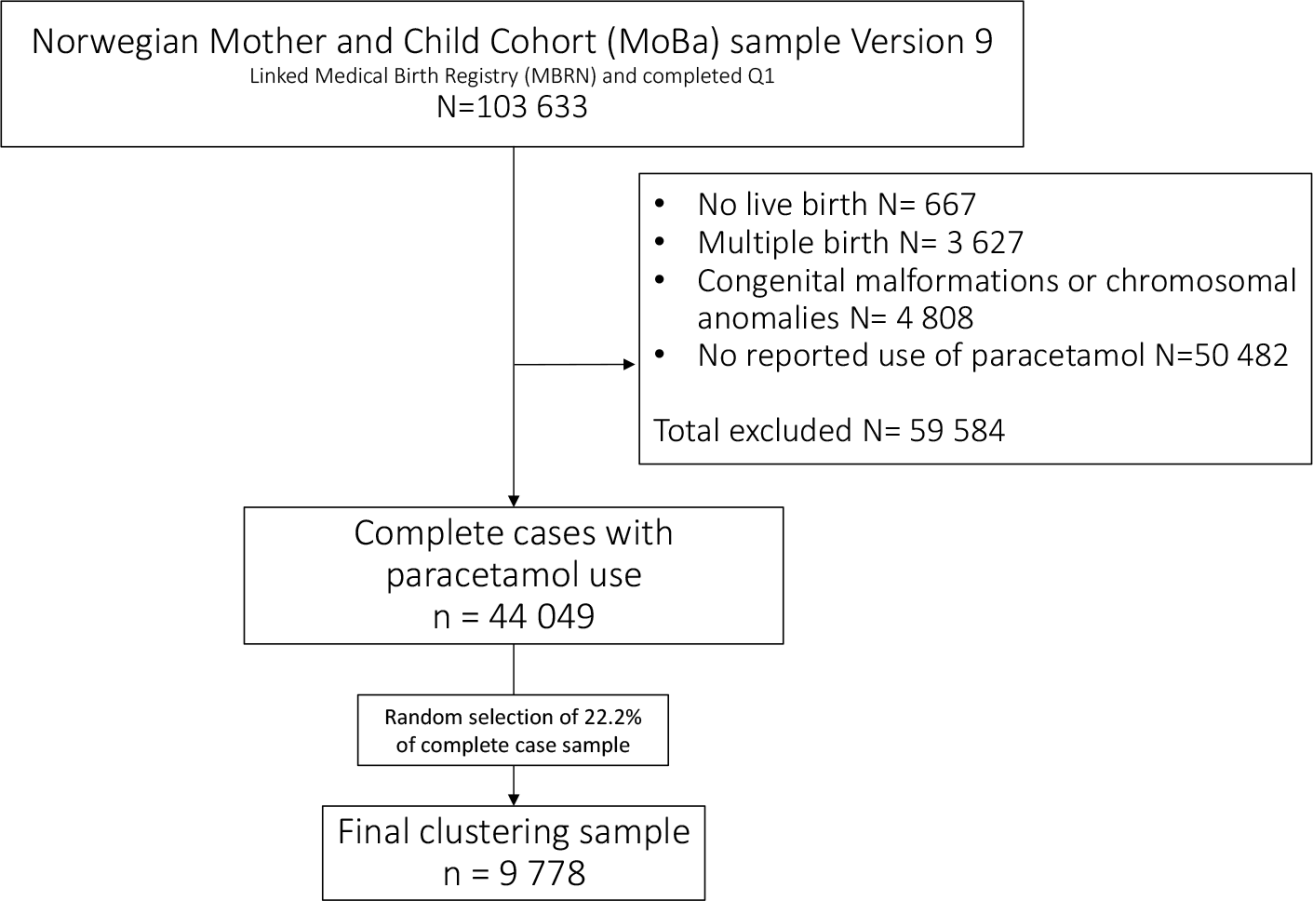
Flowchart of inclusion for the final study population.

We excluded from the study infants not born alive (n = 667), infants born with major congenital malformations or chromosomal abnormalities (n = 4808) and multiplet births (n = 3627). Women with missing questionnaires information at GW17 (Q1), GW30 (Q3), 6-months post-partum (Q4), or at 18-months (Q5) post-partum were excluded from the analyses otherwise all children exposed to paracetamol in utero were part of the study population. Because of the computational intensity of hierarchical cluster analysis, we randomly selected a sample of 9778 (circa 22% of complete cases with paracetamol use) women for the clustering procedure. A flow chart showing the exclusion criteria is provided in Fig 1.

### Paracetamol exposure

Information on exposure to medications was gathered from three questionnaires: two prenatal questionnaires (Q1 and Q3 at gestational weeks 17 and 30 respectively), and one postpartum questionnaire (Q4, 6 months after birth). 77 specific indications were listed to enhance recall. Women were asked whether they had taken a medication before or during pregnancy, and asked to indicate the period when the medication was taken. Possible responses included the 6 months before pregnancy, and ten time windows during pregnancy; weeks 0–4, 5–8, 9–12, and/or 13 or later for Q1; weeks 13–16, 17–20, 21–24, 25–28, and/or 29 or later for Q3; and from week 30 until birth for Q4. Women wrote the names of medications in a text box, and women who reported multiple medications in the same text box were considered to have been exposed to all the reported medications in all time periods. Medication use was coded according to the Anatomic Therapeutic Chemical (ATC) classification system developed by the World Health Organization. We considered medication used in each time window of the pregnancy period until birth [9]. S1 File describes how we avoided double counting of medications.

### Statistical analysis

#### Hierarchical cluster analysis

In this study women were clustered using hierarchical cluster analysis (HCA) [17, 18]. In HCA, individuals are clustered based on their distance measure, defined in our study as dissimilarity between the ATC code of the co-medications used. The algorithm for this score is presented in detail in S2 File. In brief, medications in the same ATC class are considered more similar than medications in different ATC classes, with a dissimilarity score ranging from 0 (most similar/same medication) to 3 (not similar/different medications). For example, in our study, paracetamol (ATC code N02BE01) is more similar to opioids (ATC code N02A) than to asthma medicines (ATC code R03A).

The result of HCA is a dendrogram which represents the nested grouping of individuals at which the dissimilarity changes. At the bottom of the dendrogram, each mother starts in her own cluster, and most similar clusters are merged as one moves up the hierarchy. The most similar clusters are determined by using a linkage criterion. We here used the centroid-linkage criterion [19] instead of single or average-linkage, i.e. similarity between two clusters is calculated as squared Euclidean distance between their centroids. Less distant clusters are defined as more similar and therefore merged together.

#### Optimal number of clusters

Based on the dendrogram resulting from the hierarchical clustering analysis, we identified clusters by visually inspecting and manually cutting the dendrogram based on the patterns shown by the corresponding heatmap. We also tried algorithms that automatically determine a flat list of clusters from a hierarchical clustering dendrogram based on cluster separation criteria. However, these separation criteria resulted in a very unbalanced distribution of cluster sizes that did not fit our purpose of using the cluster assignment of pregnancies for a subsequent outcome analysis.

#### Descriptive statistics

Descriptive statistics such as mean, and standard deviation of maternal characteristics such as age, education, body mass index (BMI), parity, marital status, smoking, alcohol use, folate intake during pregnancy, gestational age (weeks) at birth, birthweight and gender, and several indications such as pain (headache or migraine, pelvic girdle pain, back pain, neck pain, abdominal pain and other pain), fever and infections (genital, urinal and respiratory) were calculated for each cluster [20].

### Software

All the analyses were implemented in Python Version 2.7. [21] The cluster analysis and the silhouette score were performed using the module *scipy* [22] available in Python, while the statistical tests were implemented in R Version 3.3.2 [23].

## 3. Results

This study included 9778 pregnancies randomly selected from women who met inclusion criteria and who used paracetamol at least once during pregnancy.

### Clusters of medication use

**Fig 2:**
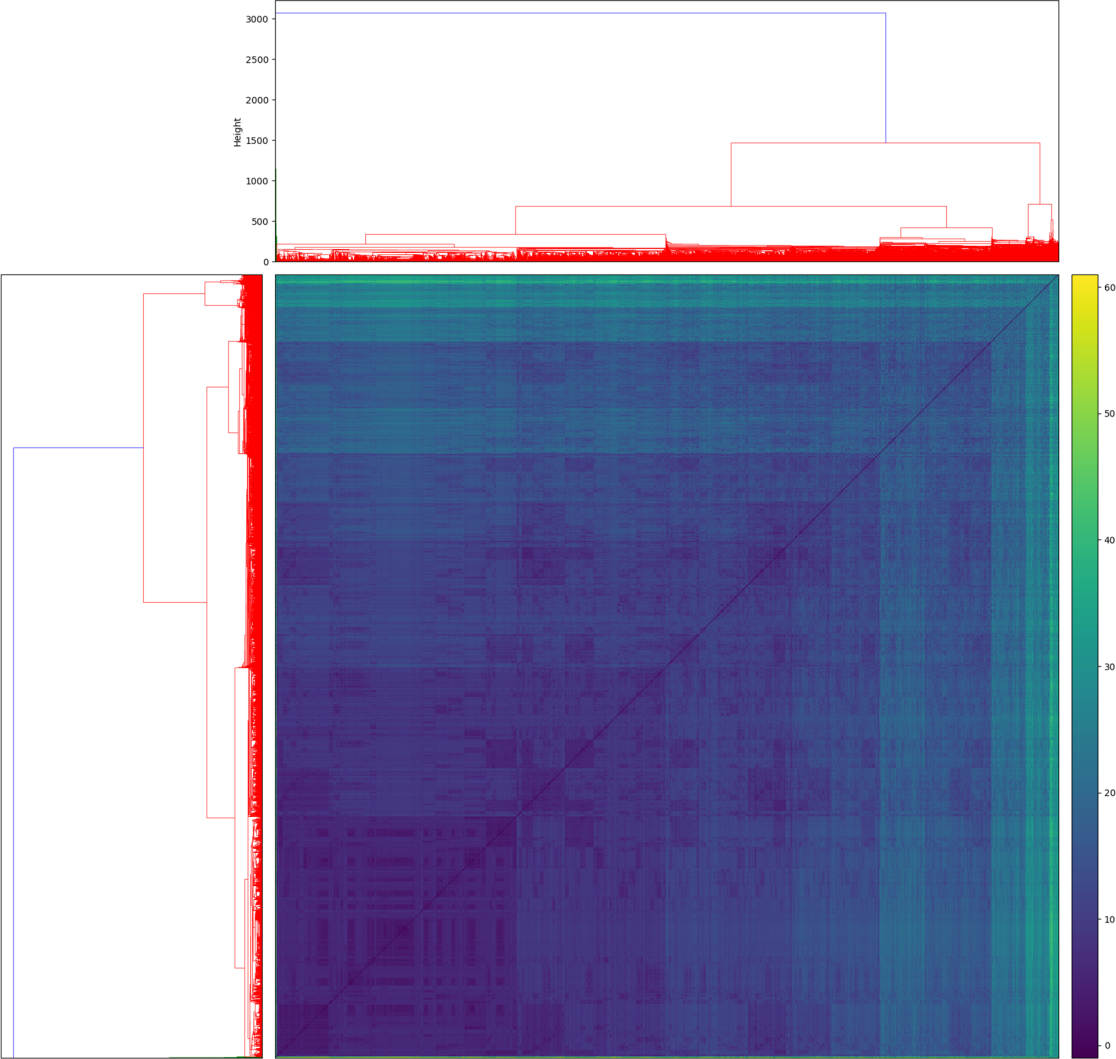
Dendrogram and heatmap of clustering results. The x-axis of the dendrogram indicates mothers exposed to paracetamol in pregnancy, while the y-axis of the dendrogram indicates at which height mothers are clustered together. The colors of the heatmap indicate the values of pairwise distance between mothers exposed to paracetamol in pregnancy. The distance values go from 0 (indicating no distance in blue) to 60 (indicating maximum distance in yellow color).

**Fig 3:**
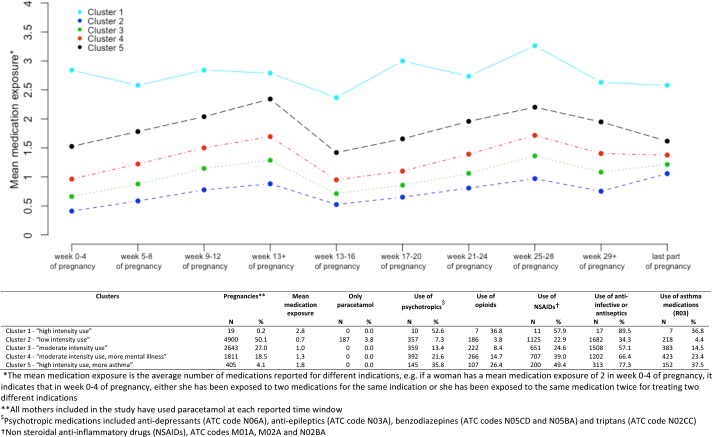
Maternal and child characteristics of exposure to different medication groups by clusters.

Fig 2 shows the dendrogram and the heatmap resulting from the hierarchical cluster analysis by using the defined customized distance measure. The visible patterns shown by the heatmap were then used for cutting the branches of the dendrogram and obtaining the clustering groups. Using this clustering approach, the sample was grouped into five clusters (Fig 3). In addition to paracetamol, which was used by all women in our sample at some point during pregnancy, anti-infectives or antiseptics were the most reported medications, followed by NSAIDs. Cluster 1(labelled “high intensity use”) was characterized by women who reported high intensity use of all medications, with a mean medication exposure of 2.8, and whose use remained high throughout pregnancy. Cluster 2 (labelled “low intensity use”) was the most populated cluster, containing 50% of all women in our sample; cluster 2 had the lowest overall medication use with an average exposure of 0.7, included 187 women used paracetamol only, with no other concomitant medications. Clusters 3 (labelled “moderate intensity use”) and 4 (labelled “moderate intensity use, more mental illness”) showed a similar pattern to cluster 2 but with higher average medication exposure; 1.0 and 1.3 respectively. Cluster 5 (labelled “high intensity use, more asthma”) included women with the highest use of asthma medications compared to other clusters and a medication exposure of 1.8.

The graph in Fig 3 shows the mean exposure to medications throughout pregnancy for each cluster, by the time window when the medications were taken. The medication exposure was usually higher in early pregnancy up to week 13, and was lower in the second and third trimesters. However, a different pattern was shown by *cluster 1* (“high intensity use”, N = 19) composed of women who only reported high intensity used of medications. Women belonging to this cluster had the highest level of medication exposure throughout the entire pregnancy period, in particular during the last trimester.

Infection was the most common indication reported for women belonging to all clusters, except for those who belonging to cluster 2 (“low intensity use”) where the most common indication reported was pain (Tables 1 and 2). Women in cluster 1 (“high intensity use”), had lower education and were more likely to use alcohol during pregnancy; women with low intensity use of medications, were more often multiparous and more likely to smoke, while women in cluster 5 (“high intensity use, more asthma”), had higher BMIs compared with other clusters.

**Table 1:** Maternal characteristics for the total cohort of 9 778 women by clusters.

		Cluster1:		Cluster2:		Cluster3:		Cluster4:		Cluster5:	
		‘high intensity use’		‘low intensity use’		‘moderate intensity use’		‘moderate intensity use, more mental illness’		‘high intensity use, more asthma’	
		N = 19	%	N = 4900	%	N = 2643	%	N = 1811	%	N = 405	%
**Maternal characteristics**^**a**^											
Maternal age	<20	0	0.0	23	0.5	12	0.5	4	0.2	0	0.0
	20–24	1	5.3	403	8.2	207	7.8	104	5.7	13	3.2
	25–29	7	36.8	1562	31.9	848	32.1	551	30.4	124	30.6
	30–34	6	31.6	2057	42.0	1102	41.7	774	42.7	194	47.9
	35–39	4	21.1	754	15.4	423	16.0	337	18.6	59	14.6
	> = 40	1	5.3	101	2.1	51	1.9	41	2.3	15	3.7
Marital status	Married/Cohabitant	17	89.5	4763	97.2	2553	96.6	1764	97.4	388	95.8
	Others	2	10.5	137	2.8	90	3.4	47	2.6	17	4.2
Maternal education	Primary/secondary school	15	79.0	3302	67.4	1809	68.4	1283	70.8	298	73.6
	University/higher degree	4	21.1	1585	32.4	823	31.1	524	28.9	105	25.9
Parity	> = 1	6	31.6	3062	62.5	1501	56.8	975	53.8	201	49.6
BMI	<18	0	0.0	64	1.3	33	1.3	31	1.7	6	1.5
	18–24	11	57.9	2525	51.5	1326	50.2	897	49.5	188	46.4
	> = 25	7	36.8	2222	45.4	1231	46.6	862	47.6	209	51.6
Folate use		12	63.2	2952	60.2	1606	60.8	1144	63.2	249	61.5
Alcohol use during pregnancy^b^		4	21.1	604	12.3	349	13.2	239	13.2	60	14.8
Smoking during pregnancy^c^		16	84.2	4148	84.7	2225	84.2	1508	83.3	333	82.2
**Health conditions**											
Pain (headache, abdominal pain, neck pain, …)		16	84.2	3220	65.7	1788	67.7	1303	71.9	316	78.0
Fever		5	26.3	920	18.8	509	19.3	423	23.4	113	27.9
Infections		19	100	2945	60.1	2133	80.7	1546	85.4	362	89.4
Mental Illness (depression, anxiety, …)		6	31.6	125	2.5	158	5.9	222	12.3	71	17.5
Asthma		6	31.6	204	4.2	347	13.1	369	20.4	145	35.8

^a^The numbers of women within each strata do not add up to the total number of women for all variables due to missing data (0.1% missing for maternal age, marital status, folate use, smoking and parity, 2% missing for BMI, 3% missing for maternal education and 5% missing for alcohol use during pregnancy)

^b^Use of one or more alcohol units reported during pregnancy

^c^Smoking daily or sometimes during pregnancy

**Table 2:** Child characteristics for the total cohort of 9 778 women by clusters.

		Cluster1		Cluster2		Cluster3		Cluster4		Cluster5	
		‘high intensity use’		‘low intensity use’		‘moderate intensity use’		‘moderate intensity use, more mental illness’		‘high intensity use, more asthma’	
		N = 19	%	N = 4900	%	N = 2643	%	N = 1811	%	N = 405	%
**Child characteristics**^**a**^											
Gender	Boy	8	42.1	2437	49.7	1283	48.5	919	50.8	194	47.9
	Girl	11	57.9	2463	50.3	1360	51.5	892	49.3	211	52.1
Weight at birth	<2500	0	0.0	99	2.0	76	2.9	65	3.6	18	4.4
	> = 2500	19	100.0	4796	97.9	2566	97.1	1745	96.4	387	95.6
Gestational week	<37	1	5.3	183	3.7	146	5.5	93	5.1	29	7.2
	> = 37	18	94.7	4695	95.8	2485	94.0	1711	94.5	375	92.6

^a^The numbers of children within each strata do not add up to the total number of children for all variables due to missing data (0.1% missing for gender, 0.2% for weight at birth and 0.5% for gestational week).

## 4. Discussion

In this paper, by using hierarchical cluster analysis (HCA) with a customized distance measure, we combined longitudinal information on timing of medication exposure together with the type of concomitant medications used, identifying five different clusters of medication exposure that were largely driven by intensity of use and thereby illustrating a novel approach of handling medication exposures in pharmacoepidemiological studies.

The analysis of data in population-based epidemiological studies tends to be dominated by regression based methods which use over-simplified exposure definitions [2, 10, 14, 15, 24–26]. In these studies, medication exposure is often categorized as short-term vs. long term exposure, ever use versus no use of medications or by trimester of use, without taking into account the entire time window when the medication is used and focusing only on level of use. Moreover, these studies often focus on the risk for exposure to a single medication, controlling for other risk factors including other medications. Our study shows that women frequently use multiple medications together, and so the HCA method we employed as pre-analysis, allows for a more real-world definition of exposure.

Cluster analysis is seldom applied to population based epidemiological studies due to its less intuitive results compared to regression-based methods, but has already shown promising results [3, 5, 27]. Hurault-Delarue and co-authors [3] used an extension of the traditional k-means algorithm to longitudinal data to simultaneously examine the intensity and timing of exposure to psychotropic medications during pregnancy, where the intensity of the medication use was determined by using the estimated DDD from the French prescription database. When data on dose intensity are not available, as was the case for our study, HCA is a flexible approach allowing for the use of a custom distance metric, for modelling similarities between medications based on their Anatomic Therapeutic Chemical (ATC) [9] classification code, as well as modelling overall similarities of concomitant medications. We have therefore chosen to use a clustering algorithm that is not dependent on quantitative variables, allowing us to directly use the ATC code for defining a customized distance measure for calculating pairwise distances between pregnancies. This way of calculating pairwise dissimilarity between mothers has the advantage of being objectively dependent on drug characteristics, such as organ or system of action, or chemical composition.

Our study is the first to apply this novel approach of customizing the distance measure for calculating pairwise distances between mothers, and provides information on the total burden of medication exposure for women during pregnancy. Multiple previous studies have investigated the association between prenatal paracetamol exposure and neurodevelopmental outcomes, and have found increased risks of ADHD or ADHD-like symptoms [10–12], delays in milestone attainment [14], conduct problems [13], and other neurodevelopmental differences [15], despite using a variety of methods to control bias from confounding, such as sibling designs [15], propensity scores [14], and negative controls [10, 12] as well as more traditional regression adjustment. In this study, all mothers have been exposed to paracetamol and future research could investigate the association between these exposure clusters and neurodevelopmental problems among children born to those mothers.

Several limitations should be considered when interpreting the results of our study. First, we performed HCA on a subsample of the total MoBa cohort, and so some rare medications may not have been captured in the clusters we identified. Second, although this study extends previous work by considering longitudinal trajectories of medication use, MoBa does not collect information on dose of medications used, and so “intensity” is based purely on the number of windows of drug exposure. Strengths of our study include the use of a large prospective cohort with contains extensive information on medication use, including high-quality data on over-the-counter medications like paracetamol. Further, exposure data are captured prior to the ascertainment of any outcomes, and recall is enhanced by using specific illnesses as prompts, [16, 28]; therefore, the exposure data is likely to be of high quality.

It is important to note that the HCA method does not purport to produce clusters that are the “true” clusters of mothers exposed to medications in pregnancy. Rather, this method describes the tendency for exposure to different drugs to be associated and non-uniformly distributed across mothers, meaning that mothers form groups of approximately shared drug exposure patterns. The hierarchical division of drug exposure pattern is objectively determined by the clustering algorithm. This makes HCA a fundamentally different approach than LCA or k-means, and based on the results, one that can be further explored and refined by researchers interested in studying naturalistic drug exposure patterns.

To conclude, by taking into account intensity, longitudinal timing and type of medication used, this study demonstrates the usefulness of hierarchical cluster analysis based on customized distance measures for defining discrete medication exposure profiles. Future studies may consider using this technique to define exposure groups when examining outcomes in children.

## Supporting information

S1 File List of similar indications. (DOCX)

S2 File Algorithm for computing co-medication dissimilarity/similarity score. (DOCX)
