# Supplementary material for: Complex patterns of concomitant medication use: A study among Norwegian women using paracetamol during pregnancy
Source: PLoS One. 2017 Dec 28;12(12):e0190101. doi: 10.1371/journal.pone.0190101 (PMC5746239; doi:10.1371/journal.pone.0190101)
Supplement: S1 File — (DOCX) [file pone.0190101.s001.docx]

List of similar indications

To avoid double counting of medications, we organized indications of each questionnaire in indication groups (following tables).

A medication used for similar indications (indications falling in the same indication group) in the same time window were all counted as one exposure. For example, someone using paracetamol for both fever and flu in the same time window would count as the same exposure. On the other hand, compounds used for different indications (different indication groups) in the same time period as well as recordings of the same medications in the same time window but for different indications (different indication groups), were all counted as two exposures. For example, someone using paracetamol for both fever and flu in the same window would count as the same exposure, while someone using it for headache in two windows or for headache and infection in the same window would count as two exposures.

Questionnaire Q1 (gestational week 17):

| **Condition** | **Indication Group** |
| --- | --- |
| fever with rash | fever |
| fever over 38.5 | fever |
| nausea | gastric illness |
| nausea with vomiting | gastric illness |
| constipation | gastric illness |
| diarrhea/gastric flu | gastric illness |
| heartburn/reflux | gastric illness |
| vaginal thrush | genitourinary infection |
| vaginal catarrh/unusual discharge | genitourinary infection |
| common cold | respiratory infection |
| throat infection | respiratory infection |
| sinusitis/ear infection | respiratory infection |
| influenza | respiratory infection |
| pneumonia/bronchitis | respiratory infection |
| edema | edema |
| abdominal pain | abdominal pain |
| pelvic girdle pain | musculoskeletal pain |
| back pain | musculoskeletal pain |
| neck and should pain | musculoskeletal pain |
| protein in urine | protein in urine |
| pregnancy itch | skin problems |
| unusual tiredness/sleepiness | sleep problems |
| sleeping problems | sleep problems |
| sugar in urine | sugar in urine |
| hay fever/pollen allergy | allergy |
| animal hair allergy | allergy |
| other allergy | allergy |
| atopic dermatitis | allergy |
| asthma | asthma |
| cancer | cancer |
| congenital heart defect | cardiovascular illness |
| other heart disease | cardiovascular illness |
| high cholesterol | cardiovascular illness |
| high blood pressure | cardiovascular illness |
| diabetes (with insulin) | diabetes |
| diabetes (not with insulin) | diabetes |
| hepatitis/jaundice | chronic gastro intestinal problems |
| gall stones | chronic gastro intestinal problems |
| duodenal/stomach ulcer | chronic gastro intestinal problems |
| Crohn's disease/ulcerative colitis | chronic gastro intestinal problems |
| celiac sprue (gluten insensitivity) | chronic gastro intestinal problems |
| other GI problems | chronic gastro intestinal problems |
| kidney stones | chronic gastro intestinal problems |
| b-12 folic acid insufficiency | Indication requiring vit/min |
| anemia/low hemoglobin | Indication requiring vit/min |
| cold sores (herpes) | cold sores |
| herpes | genitourinary infection |
| venereal warts/condyloma | genitourinary infection |
| gonorrhea | genitourinary infection |
| chlamydia | genitourinary infection |
| kidney infection | genitourinary infection |
| urinary tract infection | genitourinary infection |
| anorexia/bulimia | mental illness |
| depression | mental illness |
| anxiety | mental illness |
| epilepsy | Neurological disorder |
| multiple sclerosis | Neurological disorder |
| cerebral palsy | Neurological disorder |
| sciatica | chronic pain |
| fibromyalgia | chronic pain |
| migraine | headache |
| other headache | headache |
| arthritis | rheumatic illness |
| lupus | rheumatic illness |
| ovary/fallopian tube infection | reproductive tract problems |
| endometriosis | reproductive tract problems |
| uterine prolapse | reproductive tract problems |
| ovarian cyst | reproductive tract problems |
| myoma | reproductive tract problems |
| cervical cell changes | reproductive tract problems |
| incontinence | reproductive tract problems |
| psoriasis | rheumatic illness |
| urticaria | skin problems |
| other eczema | skin problems |
| acne/pimples (serious) | skin problems |
| hypo- or hyperthyroidism | thyroid problems |
| other long term illness | long-term Illness |

Questionnaire Q3 (gestational week 30):

| **Condition** | **Indication Group** |
| --- | --- |
| hay fever/pollen allergy | allergy |
| asthma | asthma |
| high blood pressure | cardiovascular illness |
| nausea | gastric illness |
| long term nausea and vomiting | gastric illness |
| constipation | gastric illness |
| diarrhoea, gastric flu | gastric illness |
| heartburn | gastric illness |
| vaginal thrush | genitourinary infection |
| vaginal catarrh, unusual discharge | genitourinary infection |
| bladder infection/cystitis | genitourinary infection |
| common cold | respiratory infection |
| throat infection | respiratory infection |
| sinusitis/ear infection | respiratory infection |
| influenza | respiratory infection |
| pneumonia/bronchitis | respiratory infection |
| other cough | respiratory infection |
| depression | mental illness |
| other psychological problems | mental illness |
| oedema | oedema |
| headache/migraine | headache |
| pelvic girdle pain | musculoskeletal pain |
| back pain | musculoskeletal pain |
| other muscle/joint pain | musculoskeletal pain |
| leg cramps | musculoskeletal pain |
| protein in urine | protein in urine |
| incontinence | reproductive tract problems |
| pregnancy itch | skin problems |
| unusual fatigue/drowsiness | sleep problems |
| sugar in urine | sugar in urine |
| other | other |
| fever | fever |
| fever | fever |
| fever | fever |
| other-sleeping | sedatives |
| other-sleeping | sedatives |
| other-sleeping | sedatives |
| other-sleeping | sedatives |

Questionnaire Q4 (post-partum):

| **Condition** | **Indication Group** |
| --- | --- |
| hay fever/pollen allergy | allergy |
| asthma | asthma |
| high blood pressure | cardiovascular illness |
| fever | fever |
| constipation | gastric illness |
| diarrhoea/vomiting | gastric illness |
| heartburn | gastric illness |
| cystitis | genitourinary infection |
| vaginitis | genitourinary infection |
| mastitis | mastitis |
| common cold/influenza | respiratory infection |
| sore throat/sinusitis/ear infection | respiratory infection |
| pneumonia/bronchitis | respiratory infection |
| mental health problems | mental illness |
| oedema | oedema |
| headache/other pains | pain |
| protein in urine | protein in urine |
| sugar in urine | sugar in urine |
| other | other |
