# Supplementary material for: Complex patterns of concomitant medication use: A study among Norwegian women using paracetamol during pregnancy
Source: PLoS One. 2017 Dec 28;12(12):e0190101. doi: 10.1371/journal.pone.0190101 (PMC5746239; doi:10.1371/journal.pone.0190101)
Supplement: S2 File — (DOCX) [file pone.0190101.s002.docx]

Algorithm for computing co-medication dissimilarity/similarity score

As criterion to group mothers in each cluster, we used hierarchical cluster analysis (HCA) and implemented the following function for calculating the pairwise distances between mothers:

$d\left( p_{i}, p_{j} \right)= max\{\left( \sum_{t=pregnancy window} {|D}_{p_{i}},_{t}- D_{p_{j}},_{t}| \right);CoDrugDissimilarity\left( p_{i}, p_{j} \right)\}$; with $=0.5$ and $=1$ (1)

and

$$CoDrugDissimilarity\left( p_{i}, p_{j} \right)=$$

$$=max\{\sum_{p_{i}} 3-\max\left[ CoDrugSimilarity\left( p_{iD_{1}}\ldots p_{iD_{n}},p_{j} \right) \right];\sum_{p_{j}} 3-max[CoDrugSimilarity(p_{i}, p_{jD_{1}}\ldots p_{jD_{s}})]\}$$

where *n* and *s* are the number of medications used respectively in pregnancies $p_{i}$ and $p_{j}$ for $i\neq j$. $D_{p_{i}},_{t}$ represents the use of medications for pregnancy $p_{i}$ in the time window *t* of pregnancy, while $p_{iD_{1}}\ldots p_{iD_{n}}$ and $p_{jD_{1}}\ldots p_{jD_{s}}$ indicate the specific medications $D_{1}, \ldots, D_{n}$ and $D_{1}, \ldots, D_{s}$ used in pregnancy $p_{i}$ and $p_{j}$ respectively. The last term of the formula (1) is a score which is based on the similarity between the co-medications used during the considered pregnancies.

Let us indicate with ${CoD}_{p_{i}}$ the co-medications used during the pregnancy $p_{i}$ and ${CoD}_{p_{j}}$ the co-medications used during the pregnancy $p_{j}$. ${CoD}_{p}$ is a string which contains the ATC code of the medication/medications used throughout pregnancy.

The ATC classification system divides the medications into different groups according to the organ or system on which they act and according to their chemical, pharmacological and therapeutic properties ^1^. Medications are classified in groups at five different levels. The medications are divided into 14 main groups (first level), with two therapeutic/pharmacological subgroups (second and third levels). The fourth level is a therapeutic/pharmacological/chemical subgroup and the fifth level is the chemical substance. The second, third and fourth levels are often used to identify pharmacological subgroups when these are considered to be more appropriate than therapeutic or chemical subgroups ^1^.

The ATC code of a medication was indeed used to compute the co-medication dissimilarity score as following:

$CoDrugDissimilarity\left( p_{iD_{m}},p_{jD_{l}} \right)=3-CoDrugSimilarity\left( p_{iD_{m}},p_{jD_{l}} \right)$; for m=1, 2, … n and l=1,2, … s

$CoDrugSimilarity\left( p_{iD_{m}},p_{jD_{l}} \right)= CoDrugSimilarity\left( {ATC}_{p_{iD_{m}}},{ATC}_{p_{jD_{l}}} \right)$

Set $CoDrugSimilarity\left( {ATC}_{p_{iD_{m}}},{ATC}_{p_{jD_{l}}} \right)=0$

for *k* in 1 to 5:

check if level *k,* of ${ATC}_{p_{iD_{m}}}$is equal to level *k* of${ATC}_{p_{jD_{l}}}$

if true:

then for similarity between ${ATC}_{p_{iD_{m}}}$ and ${ATC}_{p_{jD_{l}}}$add a score equal to *i**0.2

and continue to next level;

if false:

break the loop.

In this way, the maximum and minimum similarity score between two medications is 3 and 0, while the respective dissimilarity scores are 0 and 3. However, when calculating the medication similarity, we included the following exceptions:

- similarity between the two medication groups of nervous system (ATC code: N-) and, musculo-skeletal system (ATC code: M-) was set to 0.2,
- similarity between the two medication groups of analgesics (ATC code: N02-) and, anti-inflammatory and anti-rheumatic products (ATC code: M01-) was set to 0.6,
- similarity between the two medication groups of other analgesics and antipyretics (ATC code: N02B-) and, non-steroidal anti-inflammatory medications (ATC code: M01A-) was set to 1.2.

Moreover, in order to incrementally improve the performance of the clustering algorithm, we collapsed all pregnancies that had identical medication exposure values (identical sets of annotated medications and usage periods) to a single representative entity during clustering. After clustering, the dataset was expanded to include the full set of pregnancies, by including all pregnancies represented by a given entity to the cluster that the representative entity was assigned to.

References

1. Organization WH. Classifications. The anatomical therapeutic chemical classification system with defined daily doses (ATC/DDD). 2012.
